# Design and Implementation of a Secure Wireless Mote-Based Medical Sensor Network

**DOI:** 10.3390/s90806273

**Published:** 2009-08-11

**Authors:** Kriangsiri Malasri, Lan Wang

**Affiliations:** Department of Computer Science, University of Memphis, 209 Dunn Hall, Memphis, TN 38152-3240, USA; E-Mail: kmalasri@memphis.edu (K.M.)

**Keywords:** medical sensor network, neural network, elliptic curve cryptography, security

## Abstract

A *medical sensor network* can wirelessly monitor vital signs of humans, making it useful for long-term health care without sacrificing patient comfort and mobility. For such a network to be viable, its design must protect data privacy and authenticity given that medical data are highly sensitive. We identify the unique security challenges of such a sensor network and propose a set of resource-efficient mechanisms to address these challenges. Our solution includes (1) a novel two-tier scheme for verifying the authenticity of patient data, (2) a secure key agreement protocol to set up shared keys between sensor nodes and base stations, and (3) symmetric encryption/decryption for protecting data confidentiality and integrity. We have implemented the proposed mechanisms on a wireless mote platform, and our results confirm their feasibility.

## 1. Introduction

In our society, an increasing percentage of the population are aging people who have chronic illnesses such as diabetes and heart disease. In addition, more children are suffering from long-term conditions such as asthma and obesity. If these people’s health could be monitored continuously over a long period of time, physicians could detect serious health problems sooner as well as provide more accurate diagnoses and better treatment. For instance, previous studies have shown that monitoring patient data can help with early detection of conditions like heart disease [1, 2]. Moreover, medical professionals could react to situations such as strokes and asthma attacks more quickly. Current monitoring solutions, however, are not suitable for long-term purpose, as patients are typically attached to a bedside device that limits their mobility and comfort.

Several research groups (e.g., [3, 4, 5, 6, 7]) have recently integrated medical sensors with wireless *motes* for health monitoring, such as the Harvard wireless pulse oximeter [4]. A mote is low-power computing device with a wireless radio; it is typically the size of a match box or even smaller. Using the motes, these medical sensors wirelessly transmit data to base stations, where it can be accessed by physicians and nurses. This frees patients from the confinement of traditional wired sensors, allowing medical professionals to monitor their health remotely over long periods. Such *medical sensor networks* can be deployed in hospitals, long-term care facilities, and homes. Pharmaceutical companies could also use the network to monitor patients in clinical trials in order to develop better drugs. Moreover, the system can be extended to monitor the vital signs of people working in hazardous conditions, such as firefighters in a burning building, relief workers in a disaster area, and soldiers on a battlefield.

Although medical sensor networks are extremely useful and versatile, the medical data they collect is sensitive, and the privacy of such data is legally protected (e.g., the Health Insurance Portability and Accountability Act of 1996 (HIPAA) [8]). A patient’s physiological data may reveal what disease the patient has (which might be useful to parties such as insurance companies). As such, an attacker may profit financially by selling data obtained through eavesdropping. Moreover, the attacker could even cause physical harm to a patient by misreporting or spoofing the patient’s data, resulting in improper diagnosis and/or treatment. Therefore, it would be irresponsible to design and deploy a medical sensor network without adequate security mechanisms. Moreover, patients will not make use of this system if they are not convinced that their data will be kept confidential, regardless of how good the system’s performance is. To this end, our work aims to design a secure medical sensor network for health monitoring. We believe that security mechanisms must be designed into the architecture from day one rather than after the other issues are addressed, as security requires careful thoughts on where functionality should be placed and how the system components interact with one another.

Similar security issues may exist in some traditional wireless ad hoc networks and other types of sensor networks. However, a medical sensor network monitors *humans*, unlike most existing sensor networks that monitor the *physical environment*. A *human-centered* sensor network has distinct features such as the sensitive nature of the data, the mobility of sensors, and the proximity to potential attackers, all of which makes it difficult to address security.

The contribution of our work is the following. First, we identify the security requirements and challenges in a medical sensor network. Second, we propose a security architecture for wireless motes-based health monitoring based on the following security mechanisms: (1) a two-tier authentication scheme to ensure the authenticity of patient data; (2) a secure key agreement protocol based on elliptic curve cryptography (ECC) to set up symmetric keys between sensor nodes and base stations; and (3) symmetric encryption and decryption for protecting data confidentiality and integrity. Third, we have developed a prototype on the Tmote Sky platform for evaluating the security, cost, and performance of the proposed architecture and mechanisms. Note that our design could be applied in a variety of scenarios, e.g., hospitals, assisted living facilities, and homes. However, we use the terminology *patient*, *physician*, and *healthcare facility* for simplicity.

## 2. Related Work

Several research projects have been developing prototype medical sensor networks, but to provide a comprehensive security solution for medical sensor networks remains an open problem. The Code-Blue [4] project at Harvard has proposed a mote-based sensor network platform and developed an operational prototype for use in hospitals. The CodeBlue designers acknowledge the need for security in a medical environment, but addressing security requirements is not a main focus of their study. They did develop an elliptic curve cryptography (ECC) public-key implementation on Crossbow motes [9], which is much less efficient than later implementations.

The I-Living project [10] and PAS [11] propose an architecture to enable assisted living at home for elderly citizens. Although they propose that the medical sensors use IEEE 802.11 or Bluetooth for wireless communication, in contrast to the IEEE 802.15.4 radios often found on motes, the aims of the project are very similar. The authors realize the need for privacy when dealing with patient data and propose a symmetric security scheme, in which security information such as keys is stored in USB sticks that are automatically recognized once plugged into a device. However, the scheme seems to require that each device be individually configured, reducing its scalability.

ALARM-NET [6] shares many similarities with the above work, aiming to develop an architecture for wireless monitoring of residents in assisted-living facilities. ALARM-NET combines wearable medical sensors with static environmental sensors for measuring quantities such as temperature and light. Here, the authors propose that security be provided via the symmetric Advanced Encryption Standard (AES) cipher, but they are not specific about key management.

Much of the aforementioned work has made impressive progress from a hardware perspective, including development of custom medical sensors or integration of sensors with motes and other communication devices. Security has generally been covered separately and not as an integrated component of the architecture. We feel our work is complementary to these efforts, as we focus on addressing security concerns from an architectural standpoint.

There has been extensive research on wireless sensor security in general. Instead of enumerating all the prior work in this area, we highlight several classes of approaches and explain why they cannot be directly applied to long-term patient monitoring. Efficient public-key schemes have been proposed for mutual authentication between motes and base stations as well as the establishment of shared keys, e.g., [12, 13]. However, these schemes often require each mote to have its own public key, which is vulnerable to the *physical compromise of motes* (i.e., an attacker obtaining physical access to a mote) as we explain later. Another efficient approach to authentication is to use symmetric cryptography but delay the disclosure of the symmetric keys, e.g., *μ*TESLA [14]. This approach is not suitable for our application because message authentication must be instantaneous to ensure timely reaction to patient emergencies. Thus, we developed our own two-tier authentication scheme (detailed in Section 6) to account for the unique characteristics of medical sensor networks.

A large body of work has been done on key pre-distribution using symmetric cryptography (e.g., [15]). These schemes focus mainly on generating shared keys between sensor nodes. Often a pre-configured secret key is used between the base station and each mote, which is not resilient against physical compromise. In this paper, we choose ECC and asymmetric cryptography as the basis of our key agreement protocol because recent work [9, 16, 17, 18] has shown that performing ECC public-key computations on resource-constrained devices is viable. Several ECC-based key agreement protocols have been proposed, including ECDH [19, 20] and ECMQV [20]. The authenticated versions of ECDH and ECMQV both require a static public key on each mote, so they are not suitable in our system (due to the aforementioned threat of physical compromise). Consequently, we developed our own ECC-based key agreement protocol as detailed in Section 7.

## 3. Design Requirements

Our overall objective is to build a *secure* medical sensor network for health monitoring using *low-power* wireless motes. To achieve this objective, the system needs to support long-term monitoring while ensuring the privacy and authenticity of medical data. Below we elaborate on these three requirements.

First, patients with chronic diseases require **long-term monitoring**, which implies the following: (a) the sensor nodes should ideally operate for days without recharging; and (b) during the lifetime of a sensor node, it may be intentionally or accidentally turned off for various reasons, e.g. when it is out of battery power, so the design should not rely on the assumption that the sensor nodes are always on. These considerations have major influences on the design of our security mechanisms.

Second, since personal health data is extremely sensitive, a high confidence on the system’s ability to ensure **data privacy** is essential. From the healthcare providers’ point of view, a data breach would have very serious legal and financial consequences. From the users’ point of view, they may be hesitant to use the system if they are not convinced that their data will be kept confidential.

Third, **data authenticity** is very important in our system since healthcare providers rely on the data to diagnose and treat their patients as well as react to emergencies. Patients’ lives can be endangered if their data or commands from healthcare providers are intentionally changed or forged. Note that the verification of data authenticity has to be carried out as soon as data is received to ensure timely reaction to emergencies.

We emphasize that our work focuses on keeping patient data secure *as it is transferred to base stations*. The problem of data security does not stop at that point; there are still issues such as ensuring that only authorized personnel can view the data, and preventing accidental disclosure of the data. These areas are outside the scope of this particular work. We note, however, that there has been work addressing these issues; e.g., several variants of role-based access control (RBAC) schemes tailored for the healthcare industry were surveyed in [21].

## 4. Unique Challenges

### 4.1. Hardware Constraints

We have chosen to use wireless motes (such as the Crossbow MICA [22] and the Moteiv Tmote Sky [23]) as the sensor nodes in our design for several reasons. First, the motes are much smaller and lighter than other types of portable embedded devices (e.g., PDAs), making them easier to carry. Second, our specific application typically does not require complex data processing and has a low data transfer rate (e.g., 4 packets/second or less). As such, we need a device with a reasonable amount of computing power and data transmission capability, while maintaining extremely low energy consumption during sleep in order to support long-term monitoring. The Tmote Sky motes seem to have these characteristics, making them well suited for our goals. Using motes also allows for ease of prototype development, as the motes include an onboard CPU and enjoy the support of an active research community with a wealth of open-source software and tools. We note that it is certainly possible that motes with higher power CPUs could work (or even outperform the Tmote Sky) in our application, a point we plan to investigate in the future.

Unfortunately, *the resource constraints in wireless motes make it challenging to meet the abovementioned security requirements*. Motes have much lower processor speed, memory, link bandwidth, and energy supply than mobile PCs or PDAs, so our security mechanisms must be very resource-efficient. The MICA motes, for example, use an 8-bit, 8 MHz processor, and the comparable Tmote Sky platform employs a 16-bit, 8 MHz processor. Conventional security mechanisms incur high costs; some RSA operations can take over 80 seconds to run on a low-power 8-bit CPU [17]. In addition, the radio bandwidths of recent IEEE 802.15.4-compliant motes are limited to 250 kbps.

### 4.2. Considerations for Medical Sensor Networks

Security mechanisms developed for other sensor networks or in general may not be applicable to medical sensor networks due to their unique characteristics, as we explain below.

**Communication Pattern** *sensor nodes on different patients do not communicate with one another*. Instead, they communicate with one or more base stations close to the healthcare provider. Therefore, we need to protect the communication between the sensor nodes and base stations. However, most existing research focuses more on enabling secure communication among sensor nodes. Often, a simple symmetric-key mechanism is used between the sensor nodes and base stations, e.g., pre-configuring a shared secret in each sensor node and the base station. These mechanisms do not offer enough protection against *physical compromise of sensor nodes*.

**Threat Model** *physical compromise of sensor nodes* is a greater threat in medical sensor networks due to two factors: (1) *mobility*: while most other sensor networks have stationary sensors, medical sensors move with the patients and may get lost without even being noticed; (2) *accessibility*: it is relatively easy for attackers to find targets, i.e., patients in local healthcare facilities, as opposed to in remote locations such as forests or battlefields. Note that physically compromising the base stations is harder because they can be protected in secure locations. Once a sensor node is compromised, the attacker can capture any sensitive information remaining in the mote, including personal medical data and secret key material. The attacker can then use the key material to decrypt any previously recorded communication. Furthermore, the attacker can inject false data using the node. Therefore, we need to limit the amount of private information disclosed after the physical compromise as well as the damage that the attacker can cause through impersonating the patient. This has several implications for our design: (1) secret keys should be periodically updated; (2) when a node is turned off, no security material (such as a shared secret or a static public/private key) should have to be stored permanently in the node’s non-volatile memory (a pre-configured shared secret obviously does not satisfy this requirement); and (3) authentication must rely on something that the attacker cannot easily capture or forge.

## 5. Design Overview

**Figure 1.**
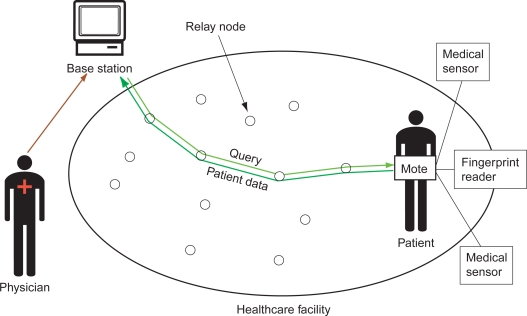
Wireless health monitoring architecture.

Figure 1 illustrates our overall architecture. Each *patient* has one wireless *mote* attached to his/her body. The mote is connected (wired) to several *medical sensors*, which take samples of the patient’s health data when they are activated. A *medical professional* issues queries for a patient’s data through one or a few *base stations*. Queries issued from base stations may activate the patient’s medical sensors or adjust their sampling frequency and other parameters. Before sending the queries, the base station verifies that the medical professional is a legitimate user with the necessary privileges to access the particular patient’s data. After the mote receives the query from the base station, it activates the appropriate sensor(s) or adjusts its parameters. It also continuously sends the resulting patient data from the sensors to the base station until the sensors are deactivated. Wireless *relay nodes* forward the queries and patient data between the base stations and the motes. Note that since our design focuses on *securing* the communication between the motes and base stations, we do not consider the routing protocol used to transfer data through the relay nodes.

### 5.1. Architectural Decisions

We have made two explicit architectural decisions that differentiate our design from previously proposed architectures such as CodeBlue [4]. These choices make it easier to secure the overall system, without unduly sacrificing the system’s functionality or performance.

First, *the motes communicate only with the base stations, not with individual medical professionals’ computers*. This is because in a large healthcare facility, it would be difficult for the motes to directly authenticate hundreds of individual users (physicians, nurses, etc.). The motes simply do not have the memory resources to maintain all this access control information. Instead, our design authenticates the users at the base stations and has the base stations issue queries to the motes on behalf of the users. Motes are configured with the base stations’ addresses and public keys for authentication.

Second, *motes do not have their own permanent public/private key pairs*, because such a key pair would have to be kept in non-volatile memory to prevent it from being lost when a mote is turned off. Since individual motes are relatively easy to physically compromise, an attacker who captures a mote would be able to decrypt all previously recorded data sent by the mote. Moreover, if the mote’s public/private key pair is used for authentication, the attacker would be able to impersonate the patient after capturing the mote.

### 5.2. Security Mechanisms

**Figure 2.**
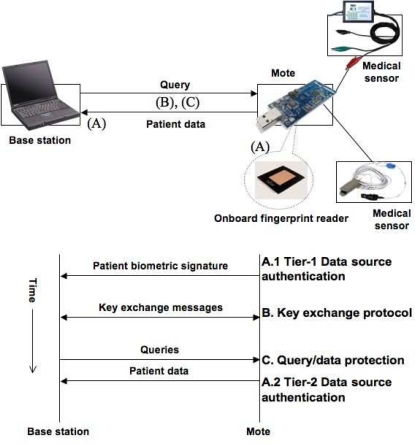
Three security mechanisms.

Our security mechanisms are summarized in Figure 2. First, we propose a *two-tier authentication scheme* based on patient biometric and physiological data, in order to handle spoofing or physical compromise of motes. We assume that each mote is connected to a small fingerprint scanner or a comparable biometric identification device. The base station first verifies the patient’s identity via a biometric signature, then checks incoming data against the patient’s profile for consistency. Our authentication scheme is resilient against spoofing and physical compromise of motes, because it is difficult for an attacker to both forge a valid patient’s biometric signature and generate physiological data that is consistent with the patient’s own data.

Second, *queries and data are encrypted and integrity protected using dynamically generated symmetric keys*, in order to defend against attacks that compromise the privacy and integrity of patient data. As mentioned earlier, motes are very resource-constrained, so it is more efficient to use pairwise *symmetric keys* shared between the base station and motes for data protection.

Third, we propose an *elliptic curve cryptography (ECC) based key agreement protocol* to allow base stations and motes to securely derive symmetric keys without any prior shared secrets. In order to handle spoofing of base stations, motes are pre-configured with base stations’ public keys so that they can establish symmetric keys only with valid base stations. If there are many base stations in the facility, we can use the *group public-key scheme* proposed in [24]. This scheme allows each base station to have its own private key, while allowing the motes to use a *single* public key for the entire base station group. In the next three sections, we present the specific design, implementation, and evaluation of each mechanism.

## 6. Two-Tier Data Source Authentication Scheme

An attacker can inject forged patient data into a medical sensor network using either his/her own motes or a compromised mote. To combat this type of attack, we propose a two-tier data source authentication scheme.

### 6.1. Design

At the first tier, each mote is integrated with a small biometric scanner to capture a unique signature for the patient. The biometric signature must be validated by a base station before communication between the base station and mote can occur. We assume that the base stations have access to a list of valid patient biometric signatures (this could be obtained from each patient upon admission to the facility), and also that the space of possible signatures is sufficiently large that a brute-force attack is infeasible. There are several off-the-shelf miniature fingerprint readers and finger vein readers that could serve as the biometric scanner. One example is the MBF200 solid-state fingerprint sensor from Fujitsu [25] (also shown in Figure 2). It is small (24 mm *×* 24 mm *×* 1.4 mm), low in power consumption (20 mA active, *<* 200 *μ*A sleep, 20 *μ*A standby), and inexpensive (about $30 per unit in bulk quantities). Moreover, it can capture a fingerprint in less than one second. The fingerprint sensor simply captures an image (which would be inefficient to transmit directly), but off-the-shelf hardware (e.g., [26]) exists that integrates the Fujitsu sensor with a small processing board to allow feature extraction from the raw image.

The first tier alone is insufficient for ensuring data authenticity because an attacker can capture a patient’s mote after the patient has been accepted into the system. Therefore, we add a second-tier authentication system to *assert the identity of a patient based solely on the sensor data being collected from that patient*. More specifically, each patient will wear the sensors for a short period of time for the base station to “learn” the patient’s profile. From then on, whenever the patient’s data deviates substantially from that profile, the base station will raise an alarm. The alarm could be caused by forged data or by a medical emergency. In either case, it is important that the patient be checked on, so issuing the alarm is warranted. This approach does not require any extra bandwidth (the data being used is sent anyway) and is completely passive from the viewpoint of the patient.

### 6.2. Implementation of Tier-2 Authentication

Our second-tier authentication mechanism requires statistical or machine learning techniques to recognize medical data as being from a particular patient. We have investigated a neural network approach for implementing this mechanism. Each patient is associated with a neural network, which is first trained on data from that patient and some reference patients. The neural network then monitors incoming data for consistency with the training data. Both the initial training and the detection mechanism are run on the base station, in order to minimize computation on the mote. Once trained, the neural network can quickly process incoming data.

**Figure 3.**
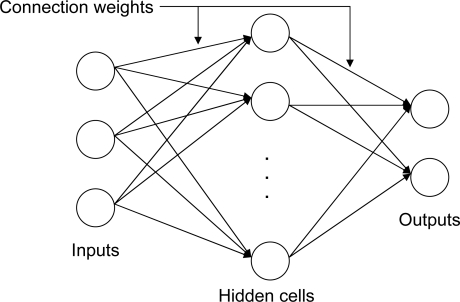
A generic three-layer feed-forward neural network.

We use a feed-forward neural network (Figure 3) that consists of a number of inputs, hidden cells, and outputs, along with a set of *connection weights* that determine how the output is computed from the input. Our current implementation uses patient electrocardiogram (ECG) signals to train the neural network, due to the high amount of identifying features that this data offers. The input to the neural network is one heartbeat of ECG data; the output is 0 or 1 (NO or YES) depending on whether the neural network believes this data is consistent with the training data.

**Figure 4.**
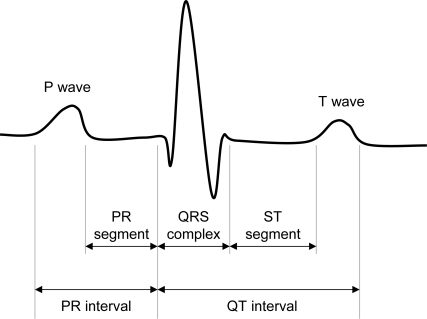
A typical ECG signal for a single heartbeat.

One issue with this approach is that raw ECG data for a single heartbeat can contain hundreds of individual samples. Using this data in its entirety is impractically complex. To address this problem we opted to extract a set of representative features from the raw ECG data to use as neural network inputs. A typical ECG signal for a single heartbeat is shown in Figure 4; it consists of a small *P wave*, followed by a large *QRS complex*, followed by a small *T wave*. We extract eight features per heartbeat: the lengths of the (1) PR interval, (2) PR segment, (3) QRS complex, (4) ST segment, and (5) QT interval; and the peak amplitudes of the (6) P wave, (7) QRS complex, and (8) T wave.

### 6.3. Evaluation of Tier-2 Authentication

As we do not yet have access to real ECG sensors, we used publicly available data from the long-term ST ECG database at MIT’s PhysioWeb [27] for developing our neural networks. Each patient’s ECG data covers a period of 21–24 hours. We set aside some of these patients as a “reference set.” For each of the remaining patients, a training data set was constructed consisting of the first 2000 heartbeats from that particular patient’s record (associated with a neural network output of 1) and the first 2000 heartbeats from each of the patients in the reference set (associated with a neural network output of 0).

We performed some experimentation with different neural network setups by varying the number of reference patients used in training as well as the features extracted from each beat of ECG data. The results in Table 1 suggest that increasing the number of reference patients potentially has the greatest returns on performance. For subsequent experiments, we chose the setup with the best overall performance for the training of each patient’s neural network, i.e., 9 reference patients and a four-layer feed-forward neural network model with 8 input cells, 2 layers of 15 hidden cells, 1 output cell, and a log sigmoid activation function on the hidden layers.

Table 2 shows how well the trained neural networks could classify the training data from 10 patients (patients are labeled *P*_1_*, ...P*_10_, and reference patients are labeled *R*_1_*, ...R*_9_). The number corresponding to (*P_i_*, self) indicates the percentage of training samples (heartbeats) from patient *P_i_* that were correctly classified as from that patient. The number (*P_i_*, *R_j_*) indicates the percentage of training samples from reference patient *R_j_* that were correctly classified as *not* being from patient *P_i_*. For example, the fourth row of Table 2 indicates that (1) the trained network for patient *P*_4_ was able to correctly classify 99.85% of the training inputs from *P*_4_ as being from *P*_4_ (the first value); and (2) it was able to classify 100% of the training inputs from reference patients *R*_1_, *R*_2_, *R*_4_*...R*_9_, and 99.93% of the training inputs from *R*_3_, as *not* being from *P*_4_. Overall, the neural networks learned the training data very well, with an average accuracy of 99.8%.

Next, we use each trained neural network to classify the validation data (i.e., 10,000 heartbeats not used in training) from each of the non-reference patients. As shown in Table 3, the neural networks give accurate results in most cases. For example, the fourth row shows that patient *P*_4_’s neural network was able to correctly classify the validation data from patients *P*_1_ through *P*_6_ and *P*_8_ through *P*_10_ almost 100% of the time, but was less accurate with *P*_7_. The average validation accuracy for the neural networks over all patients was 87.7%.

To see how well these results might generalize, we also expanded the neural network training to a larger set of data from the long-term ST database (26 patients *P*_1_ ... *P*_26_, with the same set of reference patients *R*_1_ ... *R*_9_). We found that the overall effectiveness of the neural networks on the larger set was comparable to the figures presented here, with an average accuracy of 99.7% on the training data and 91.5% on the validation data. Due to space constraints we do not include the detailed results.

It is important to note that perfect classification is *not* required at all in our scheme since our goal is to detect suspicious data. We just need to raise an alarm when a certain percentage of incoming data deviates from a patient’s profile. The above results suggest that the alarm threshold can be set to a relatively small number (e.g., 10–30%) with a low false positive and low false negative rate. In fact, with a threshold of 25%, we would have 0 false positives and only 1 false negative (*P*_1_ could be considered *P*_9_) in Table 3. One may further lower the threshold to reduce the false negative rate, with the possibility of increasing the false positive rate. As long as the false positive rate is relatively low, this may be a desirable trade-off: a false negative could have serious consequences, e.g., neglecting false data or missing an emergency situation, while a false positive simply leads to a re-examination of the patient.

## 7. ECC-based Key Agreement Protocol

We propose a key agreement protocol that each mote uses to securely derive symmetric keys with the base station at the beginning of their communication. We use *elliptic curve cryptography*.

### 7.1. Design

We first present some background on ECC and then describe the design of our key agreement protocol and key update protocol.

#### ECC Background

An *elliptic curve* is of the form *y*^2^ = *x*^3^ + *ax* + *b*. When defined over a finite field, all the points on the curve (*x, y*) and the parameters *a* and *b* are limited to elements of the underlying field. A common class of finite fields used in ECC are *prime fields GF* (*p*), where *p* is a large prime number; the elements of this field are the integers in [0, *p* − 1]. There are three basic operations that can be performed on elliptic curve points: *addition* of two points *P* + *Q*, *doubling* of a point (addition to itself) 2*P*, and *scalar multiplication nP*, where *n* is an integer. Algebraic formulae exist for both addition and doubling. Scalar multiplication is the repeated application of addition and doubling: *nP* = *P* + *P* + ... + *P* for *n* times. To use ECC, two nodes *A* and *B* agree on what elliptic curve to use and a base point *G* on the curve; this information is not secret. Node *A* can generate a large random number *q* as its private key and derive its public key *Q* by using *Q* = *qG*. If *q* is large, it is hard for an attacker to derive the private key *q* from the public key *Q* even if the attacker knows *G*, due to the difficulty of the *elliptic curve discrete logarithm problem* (ECDLP).

**Figure 5.**
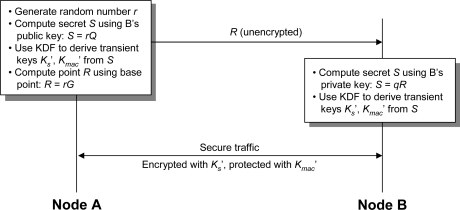
The elliptic curve integrated encryption scheme (ECIES).

We use the *Elliptic Curve Integrated Encryption Scheme* (ECIES [19]), a public-key encryption scheme based on DHIES [28], to secure the *key agreement protocol messages* between a mote and a base station. DHIES (and ECIES) is secure against adaptive chosen-ciphertext attacks (see [28] for the security proof). The ECIES procedure is summarized in Figure 5 and described below. To protect the messages between node *A* (a mote) and node *B* (base station) against eavesdropping and modification, node *A* generates a random number *r* and computes a secret *S* = *rQ*, where *Q* is node *B*’s public key. Node *A* then uses a key derivation function (KDF) to generate two *transient keys* from *S*: a symmetric encryption key *K^′^_s_* and a MAC (message authentication code) key *K^′^_mac_*. Node *A* also computes the point *R* = *rG*, which is sent in the clear to node *B*. Node *B* can derive the secret *S* using its private key *q* and the point *R: S* = *qR* = *q*(*rG*) = *r*(*qG*) = *rQ*. Node *B* then uses the same KDF to derive *K^′^_s_* and *K^′^_mac_* from *S*. The transient keys are then used to encrypt and integrity-protect the messages between *A* and *B*.

#### Key Agreement Protocol

**Figure 6.**
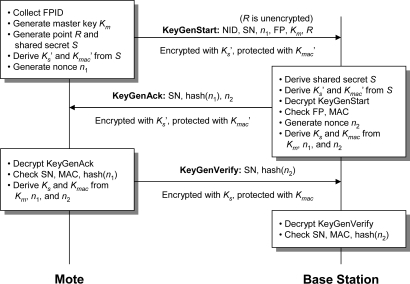
ECC-based key agreement protocol.

In our scheme, each base station has a private key *q* and a public key *Q* = *qG*, where *G* is a chosen base point on the elliptic curve. The public key *Q* is pre-configured in the motes. As mentioned before, motes do not have their own public/private keys. To securely derive symmetric keys between a mote and a base station (called *session keys*), we use the following protocol involving three messages (Figure 6).

When a mote is first attached to a patient, the patient uses the biometric scanner on the mote to activate the key agreement protocol. For illustration, we assume that a fingerprint reader is used to obtain a biometric signature, but other types of biometric information would also work. Once the mote obtains the patient’s fingerprint (*F P*), which includes a set of features extracted from the raw fingerprint image, it generates a master key *K_m_* that will be used to derive the session keys for end-to-end query/data encryption. It also generates a session number *SN* that uniquely identifies this particular communication session, and a nonce *n*_1_ for deriving the session keys. The mote then sends a *KeyGenStart* message *securely* to the base station using ECIES. This message contains the mote’s ID (*NID*), the patient’s *F P*, the master key *K_m_*, the session number *SN*, and the nonce *n*_1_. Appended to *KeyGenStart* is the unencrypted point *R* needed for ECIES.

The base station decrypts *KeyGenStart* and verifies the authenticity of this patient using the finger-print. Then the base station generates a nonce *n*_2_, and it uses *K_m_*, *n*_1_, and *n*_2_ to derive the session keys that will be shared with the mote — the symmetric encryption key *K_s_* and the MAC key *K_mac_* for data/query transfer. The nonces *n*_1_ and *n*_2_ not only allow frequent update of the session keys, but also protect the protocol from replay attacks. Note that our protocol is also secure against man-in-the-middle attacks because the attacker would need the base station’s private key and the patient’s *F P* to launch such an attack.

After processing the *KeyGenStart* message, the base station sends back a *KeyGenAck* message to the mote containing *SN*, *n*_2_, and a one-way hash of *n*_1_, encrypted and integrity-protected using the transient keys derived from ECIES. We opt to use a hash of *n*_1_ rather than *n*_1_ directly to reduce the information that an attacker will have even if the attacker is able to decrypt this message.

The mote decrypts *KeyGenAck* and verifies *SN* and the hash of *n*_1_ to prevent replay attacks. Afterwards, the mote uses *K_m_*, *n*_1_, and *n*_2_ to derive the session keys *K_s_* and *K_mac_*, which should match those generated by the base station. For the base station to verify that the mote has received the *KeyGenAck* message and that the mote generated the session keys *K_s_* and *K_mac_* correctly, the mote sends a *KeyGenVerify* message to the base station containing *SN* and a hash of *n*_2_. This message is encrypted with *K_s_* and integrity-protected with *K_mac_*. The base station decrypts *KeyGenVerify* and verifies that all the values are correct. If so, data transfer may commence using *K_s_* to encrypt/decrypt messages and *K_mac_* to compute a keyed MAC for each message.

Our key agreement protocol differs from the handshakes in SSL/TLS in the following ways: (1) we use only three protocol messages to set up the session keys, as message transmissions consume a significant amount of energy; (2) since motes do not have their own public/private keys, we incorporate the patient biometric signature in *KeyGenStart* as a means of authenticating the mote to the base station; (3) the nonces are encrypted in our protocol for more protection; and (4) in addition to the patient biometric signature, we have several other built-in measures to limit what an attacker can do with a compromised mote, as discussed below.

#### Key Update Procedure

The base station and the mote periodically update the session keys *K_s_* and *K_mac_* to limit the amount of private data that can be recovered in case the keys are compromised. To update the session keys, they simply exchange new values of *n*_1_ and *n*_2_ and rerun the key derivation function using the existing master key *K_m_* and the new nonce values. This allows the session keys to be updated without having to undertake the expensive public-key operations in the full key agreement protocol. We omit the details due to space constraints.

#### Further Considerations for Physical Compromise of Motes

We enhance the above mechanisms with the following measures to limit what an attacker can do with a compromised mote. Suppose that an attacker has been eavesdropping on the communication between the mote and the base station. We have two measures to bound the amount of private information that the attacker can recover from earlier captured sensor data. First, once the key update procedure produces a new session key, the previous session’s keys (*K_s_*, *K_mac_*) and nonces (*n*_1_, *n*_2_) are erased from memory. Therefore, the attacker can only decrypt the data that has already been sent in the *current* session. Second, if the base station and the mote have not communicated for a long period of time (e.g., if the patient removes the mote or if the mote is inadvertently lost), the master key *K_m_* and its derived keys expire and are removed from memory. The attacker will not obtain any session keys in this case. Note that when the master key expires, the entire key agreement protocol must be re-run to resume communication between the mote and base station. The idle time before *K_m_* expires can be tuned to fit specific security needs, with a shorter idle time providing added security at the expense of greater patient inconvenience (since the biometric security procedure would have to be reactivated) and greater computational load for key agreement.

Suppose that the attacker wants to send forged data using the mote. If the mote has been idle for too long and the master key has expired, the attacker needs to use a valid fingerprint to activate the key agreement protocol. To prevent this from happening, we remove *F P* from the mote’s memory as soon as the *KeyGenAck* message is received, so it is difficult for an attacker to obtain a valid patient’s biometric information. On the other hand, if the master key is still valid, the attacker can use the current session key and establish new session keys through the key update procedure. However, the attacker will most likely be detected by the base station via the second-tier authentication scheme, as the forged patient data will not match the existing profile.

### 7.2. Implementation of Key Agreement Protocol

Our development environment is TinyOS 2 [29] running on the Moteiv Tmote Sky platform, which features a 16-bit, 8 MHz Texas Instruments MSP430 processor with 48 KB of program ROM and 10 KB of RAM. Program code in this environment is written in nesC (networked embedded systems C), a dialect of C designed for use with TinyOS. (As of late 2007, Moteiv has changed its name to Sentilla and has discontinued production and support of its Tmote product line in favor of a new hardware platform designed for Java applications. However, the new platform is backwards-compatible with the Tmote Sky. Furthermore, Crossbow Technology still offers its TelosB mote for sale, which is functionally identical to the Tmote Sky. Both the TelosB and Tmote Sky remain popular in the research community.)

Our basic ECC operations are based on North Carolina State University’s TinyECC 1.0 distribution [16]. We implemented ECIES for secure key agreement, using the elliptic curve secp160r1 defined over a 160-bit prime field as recommended by [30]. We use 160-bit private keys, 320-bit public keys, and a 160-bit random number *r* in ECIES. We then implemented our key agreement protocol, using a 32-bit session number (*SN*), 48-bit patient fingerprint (*F P*), 128-bit master key (*K_m_*), 128-bit transient and session keys (*K^′^_s_*, *K^′^_mac_*, *K_s_*, *K_mac_*), and 64-bit nonces (*n*_1_, *n*_2_). The resulting *KeyGenStart*, *KeyGenAck*, and *KeyGenVerify* messages are 56, 52 and 32 bytes long.

Due to its customizability and ease of implementation, we chose the RC5 block cipher in ciphertext stealing (CTS) mode for the symmetric encryption in ECIES, using the recommended parameters of 64-bit blocks, 128-bit keys, and 12 rounds. For the hash function we chose SHA-1 [31], which allowed us to save code space by using SHA-1 based algorithms for both the message authentication code function (for integrity checking) and key derivation function. We implemented HMAC-SHA-1 [32] and PBKDF1 [33], respectively.

### 7.3. Evaluation of Key Agreement Protocol

Our compiled code on the Tmote uses 30 KB of ROM and 4.4 KB of RAM, which leaves reasonable space for other applications. We note that most of this memory requirement comes from TinyECC, which includes numerous optimizations to speed up the ECC operations. As indicated by Liu and Ning [16], these optimizations can be selectively disabled to reduce the code size at the cost of increased computation time.

**Figure 7.**
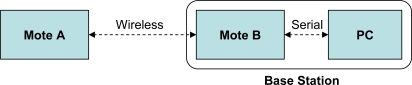
Experimental setup with two motes and a PC.

We timed our protocol which ran on two motes and had a laptop PC (2.66 GHz Intel Pentium 4) as a base station. As illustrated in Figure 7, mote *A* acts as a sender, while mote *B* serves as the interface between the sender and the PC by receiving *A*’s wireless packets and forwarding them over a serial connection (emulated via a physical USB connection). Mote *B* essentially serves as a bridge between the sending mote and the PC, as the PC lacks a native way of wirelessly receiving data from a mote. Mote *A* took 7075 ms to generate and send *KeyGenStart*, and 123 ms to check *KeyGenAck* and send *KeyGenVerify*. The base station took 77 ms to check *KeyGenStart* and send *KeyGenAck*, and 13.3 ms to check *KeyGenVerify*. Virtually all of the time required to generate and send *KeyGenStart* is due to two ECC scalar point multiplications (computing the points *rQ* and *rG*, as described previously).

Although a CPU time investment of over 7 s may seem intensive, we note that the key agreement protocol only has to be run once to establish the initial symmetric keys, and once each time the keys expire. We explain in Section 8.5 that this periodic investment has minimal effect on the mote’s battery life.

## 8. Query/Data Protection Mechanism

Queries and data are encrypted and integrity-protected using the session keys established through the key agreement protocol. To prevent replay or injection of messages, we use both a *session number* (*SN*) and a *message sequence number* in query and data messages. The session number is randomly changed whenever the keys are updated (if the master key has expired, the session number is re-generated by the sending mote as part of re-running the entire key agreement protocol; if only the session keys are being updated, the session number is updated via the nonce exchange procedure). The base station needs to keep a list of recently used session numbers in order to detect replay attacks. This list can be stored efficiently using a bloom filter. The base station and the mote also increase their message sequence numbers for each new query or data message. Received messages with an unexpected session number or sequence number are discarded. Note that it is difficult for an outside attacker to guess a session number in the first place.

To save code space, we use the same symmetric cipher and MAC function in the query/data protection mechanism as in the key agreement protocol discussed previously (RC5 in ciphertext stealing mode and HMAC-SHA-1, respectively).

### 8.1. Overhead of Symmetric Encryption/MAC

**Figure 8.**
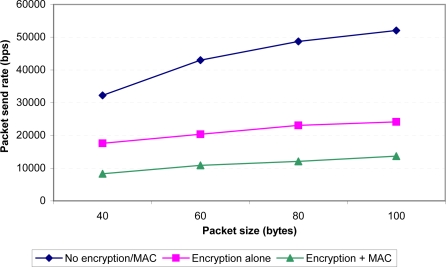
Throughput as a function of packet size and encryption.

To get an idea of the performance impact of performing symmetric encryption/MAC on every data packet, we conducted a simple experiment in which mote *A* (see Figure 7) sent 2000 packets over the air as rapidly as possible. We varied the packet size from 40 bytes to 100 bytes (near the upper limit of packet size for our platform) and measured the throughput of the system. As illustrated in Figure 8, using encryption and integrity checking does lower the throughput from 31–53 kbps to 8–14 kbps (depending on packet size). We also directly measured the time required to perform the MAC and encryption operations on a single 100-byte packet and we found that this consumes about 44 ms of CPU time. However, we feel that this is a reasonable price to pay for the added security. Moreover, the lower throughput is still sufficient for a practical deployment, as we explain later.

### 8.2. Simulated Real-World Deployment

We then set up a more realistic network with multiple senders and one receiving mote, with a fixed sending rate of *four* 100-byte packets per second (both encryption and MAC are enabled). We chose this sending rate (400 bytes/s per patient) based on the following considerations. The ECG data in the long-term ST database was sampled at 250 Hz. Allotting 2 bytes for each sample, this results in 500 bytes per second of raw data. However, we found that 500 bytes of raw ECG data can be reduced to about 260 bytes using S-LZW [34], a compression scheme specifically designed for the resource constraints of wireless motes. Compression would thus allow ample space for ECG data as well as other types of patient data to be sent. Note that other patient data (e.g., body temperature) could be sampled at a much lower rate than the ECG data.

**Figure 9.**
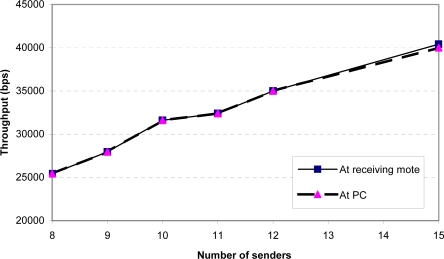
Aggregate throughput at receiving mote and PC as a function of the number of senders (sending rate = 4 packets/sec).

**Figure 10.**
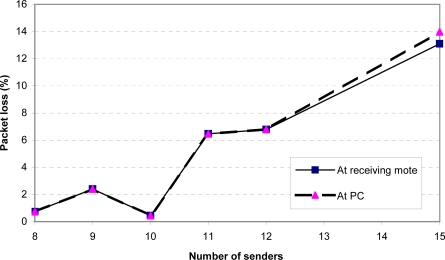
Packet loss at receiving mote and PC as a function of the number of senders (sending rate = 4 packets/sec).

Figures 9 and 10 show the throughput and packet loss as measured at the receiving mote (mote *B* in Figure 7) and at the PC. Any difference between the two curves in each figure indicates that packets were dropped by the serial connection between the receiving mote and the PC. We found that a single receiving mote is able to reliably handle 10 senders, with a packet loss of under 1%. At 11 senders and above, the receiving radio is unable to keep up with the incoming packets.

### 8.3. Stress Test

**Figure 11.**
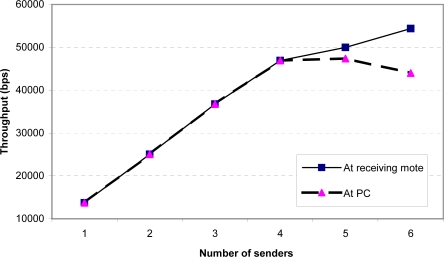
Aggregate throughput at receiving mote and PC as a function of the number of senders (maximum possible sending rate).

**Figure 12.**
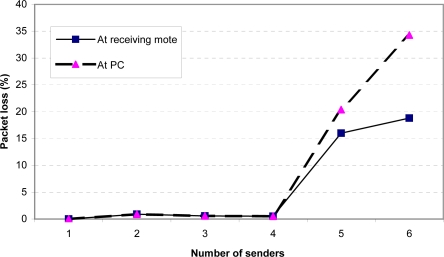
Packet loss at receiving mote and PC as a function of the number of senders (maximum possible sending rate).

For the stress test, we modified the senders to send 100-byte packets with MAC/encryption as quickly as possible, at about 17 packets per second. Note that in practice the senders are unlikely to send their sampled data at such a high rate, due to energy consumption concerns. We wanted to evaluate this scenario solely to see how the system would perform at its hardware limits. Figures 11 and 12 show the throughput and packet loss. These two figures demonstrate that a single receiving mote is able to reliably handle data from up to 4 senders sending at their maximum rate. Beyond this point, the receiving radio is unable to keep up with the incoming packets, resulting in significant packet loss. Additionally, when there are more than 4 senders, the serial connection between mote *B* and the PC also becomes a bottleneck, resulting in a higher loss rate observed at the PC than at the receiving mote. To address the radio packet loss issues, we attempted using the PacketLink reliable transmission layer in TinyOS. However, we found that this further lowered throughput as the high loss rate resulted in many retransmissions. Overall, we feel that the MAC layer being used in TinyOS can be improved to more gracefully handle high loads; we plan to further investigate this in the future.

### 8.4. Improving Base Station Scalability

**Figure 13.**
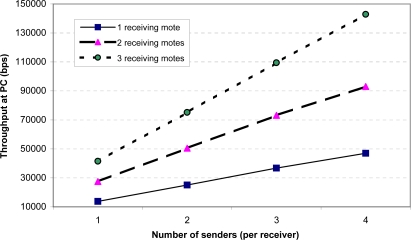
Aggregate throughput at PC as a function of the number of senders and receivers (maximum possible sending rate).

The above results may seem to suggest that a single base station can support only a very small number of sending motes. However, the number of motes reliably handled by a base station can be improved significantly simply by attaching more than one receiving mote (mote *B* in Figure 7) to the PC. Each receiving mote is set to a different wireless channel and handles incoming packets only from motes sending on that channel. To demonstrate the feasibility of this approach, we repeated the above stress test with two and three receiving motes. As shown in Figure 13, we found that the base station’s ability to handle multiple sending motes scales very well with the number of receiving motes attached. The overall throughput increases linearly with additional receiving motes. With four senders per receiver, for instance, a single receiving mote allows a throughput of about 46.9 kbps; this increases to 93.0 kbps and 142.9 kbps for two and three receiving motes, respectively. We also observed that the packet loss remains low (on the order of 1%) with the additional receiving motes.

### 8.5. Energy Considerations

A complete energy profile for the sending mote must consider the processor, the radio, and the attached medical sensors. Of these components, the processor is used heavily during the initial ECC operations and in the encryption/MAC of outgoing packets. As discussed previously, the ECC key agreement consumes 7198 ms of CPU time on the mote, while performing encryption/MAC on a single 100-byte packet consumes 44 ms of time.

The radio and the sensors are used periodically to sample patient data and forward it to the base station. Based on the previous experiments, the time required for the radio to send a single a 100-byte packet is about 15 ms. In addition to the radio, the mote’s batteries would have to be used to power the attached biometric scanner and medical sensors. Because we have not yet integrated these sensors onto our motes, it is difficult for us to quantify their energy requirements. However, we note that the scanner is needed only once each time the key agreement protocol is run. Meanwhile, the medical sensors do not have to be continuously working; they might operate in a low-power sleep mode, being activated only when required to sample the patient’s data.

To get a rough estimate of energy consumption in a real-world deployment, we made the following assumptions: (1) the ECC key agreement protocol will be run once per 24 hours; and (2) the mote will send patient data at the rate of four 100-byte packets per second. The Tmote Sky draws 5.1 *μ*A with the processor on standby and radio off, 1.8 mA with the processor alone on, and 19.5 mA with the processor on and the radio transmitting [23]. For the envisioned processor/radio usage, the mote’s AA batteries (using a conservatively low estimate of 500 mAh) would be sufficient for about 14 days of operation. The majority (78.5%) of the power requirement comes from the radio, with 21.2% from CPU use during encryption/MAC and less than 1% from CPU use during the ECC key setup and idling.

## 9. Conclusion and Future Work

We have presented the design and implementation of a comprehensive security solution for medical sensor networks. Our initial results on the patient authentication using ECG data are promising, while our implementation on the Tmote Sky demonstrates reasonable computational overhead for our mechanisms.

Our future plans include improvements to both the theoretical and implementation aspects of the system. We have not yet addressed some security requirements that would be important in an actual deployment. In particular, these security issues deserve attention:

**Base stations** - Our work assumes that the base stations in the architecture are secure. Although we feel that base stations are less likely than motes to be compromised, we should nonetheless develop a contingency plan should an attacker gain access to one or more base stations. Furthermore, we currently assume that base stations’ public keys are *pre-configured* in the patient motes. However, we have not yet developed a scheme to allow scalable and secure updates to these public keys.
**Denial-of-service attacks** - An attacker may simply flood the wireless channel with meaningless transmissions, rendering the motes unable to send any data. Some work has already been done on addressing DoS attacks in wireless sensor networks; Raymond and Midkiff [35] provide a survey. We hope to integrate some techniques from the literature into our system.
**Attacks on the relay nodes** - Rather than targeting base stations or patient motes, an attacker might focus on the relay nodes in the system. This might include conducting a DoS attack against or physically capturing relay nodes, both of which would result in a routing “black hole” in the system. We note that this might be addressed by selecting an appropriate flexible routing protocol, which (as mentioned in Section 5) we do not consider in this work.
**Random number generation** - In our scheme, the session keys are derived from a secret *S* = *rQ*, where *Q* is the public key of the base station and *r* is a random number generated by the sending mote. Even with no knowledge of *r* itself, an attacker who physically captures a mote might gain knowledge of the seed used to generate *r*. To combat this, we might have the sending mote use patient biometric data in the random number generation, but this is not yet something that we have analyzed in detail.
  Implementation-wise, we plan to evaluate the base station’s neural network mechanism using live data streams from the motes, optimize our neural network performance, integrate motes with actual medical sensors and fingerprint readers, and possibly consider a more powerful mote platform such as the Intel iMote2. The iMote2’s processor is about an order of magnitude faster than that of the Tmote Sky, which may potentially result in a more efficient system (as the processor can quickly perform its work and resume a low-power sleep mode). Ultimately, we hope to conduct a study on actual patients to demonstrate the real-world feasibility of our system. Even further in the future, we may consider using a body sensor network (which can include implanted as well as wearable wireless sensors) on the patient rather than connecting all sensors to a single mote.

## Figures and Tables

**Table 1.** Comparison of neural network setups.

**Structure**	**Features**	**Ref. Patients**	**Accuracy**

8×20×1	8	5	69.79%
8×20×1	8	9	81.86%
14×20×1	14	5	80.07%
14×20×1	14	9	84.44%
8×15×15×1	8	9	91.31%
14×15×15×1	14	9	89.66%

Structure refers to the number of input, hidden, and output cells in the neural network.

Ref. Patients refers to the number of reference patients.

The accuracies provided represent the average validation accuracy over *all* patients.

**Table 2.** Neural network results on training data.

**Patient**	**Self**	*R*_1_	*R*_2_	*R*_3_	*R*_4_	*R*_5_	*R*_6_	*R*_7_	*R*_8_	*R*_9_

*P*_1_	99.55	100	99.96	99.96	100	99.99	100	99.98	100	99.85
*P*_2_	99.95	100	100	100	100	99.99	100	100	100	100
*P*_3_	98.77	100	99.99	99.85	100	99.73	100	99.93	99.99	99.97
*P*_4_	99.85	100	100	99.93	100	100	100	100	100	100
*P*_5_	99.93	100	100	99.99	100	99.95	100	100	100	100
*P*_6_	99.27	100	100	99.77	99.99	99.9	100	99.91	99.99	100
*P*_7_	99.78	100	100	99.95	99.99	99.99	99.99	99.99	100	100
*P*_8_	99.42	99.98	100	99.78	100	99.74	100	100	99.99	99.99
*P*_9_	94.11	99.87	99.85	99.6	100	96.8	99.94	99.67	99.95	99.67
*P*_10_	95.07	99.94	99.78	99.8	99.95	97.9	100	99.49	99.93	99.65

**Table 3.** Neural network results on validation data.

**Patient**	*P*_1_	*P*_2_	*P*_3_	*P*_4_	*P*_5_	*P*_6_	*P*_7_	*P*_8_	*P*_9_	*P*_10_

*P*_1_	93.85	98.74	99.55	51.82	99.44	83.5	87.97	86.12	80.65	95.05
*P*_2_	100	98.63	99.99	99.8	90.7	100	97.45	99.66	99.95	97.33
*P*_3_	62.75	99.91	85.86	95.29	91.17	69.97	99.67	76.05	85.71	93.12
*P*_4_	99.73	99.63	99.88	98.58	99.83	99.65	87.32	96.6	97.75	99.63
*P*_5_	99.83	82.85	97.77	99.84	91.41	98.66	99.99	93.72	98.54	98.78
*P*_6_	58.31	99.78	95.62	76.4	91.08	90.24	65.29	70.51	91.47	96.14
*P*_7_	99.95	93.62	99.98	78.2	98.82	93.97	82.67	99.92	99.61	99.6
*P*_8_	91.72	93.3	71.24	43.91	88.33	88	92.45	93.91	94.08	80.99
*P*_9_	14.68	99.94	98.14	26.52	92.51	87.26	86.35	80.59	79.96	75.46
*P*_10_	63.92	51.94	92.97	31.04	84.21	91.64	88.8	76.26	84.8	87.8
